# Dural arteriovenous fistula with internal jugular venous drainage in the foramen magnum: A case report

**DOI:** 10.1097/MD.0000000000043573

**Published:** 2025-07-25

**Authors:** Rui Shang, Yu-Hu Ma, Si-Hao Li, Ting Wang, Sen Li, Hai-Tao Hu, Seidu A. Richard, Chang-Wei Zhang

**Affiliations:** a Department of Neurosurgery, West China Hospital, Sichuan University, Chengdu, Sichuan Province, China; b Institute of Neuroscience, Third Affiliated Hospital, Zhengzhou University, Zhengzhou, China; c Department of Biochemistry and Forensic Sciences, School of Chemical and Biochemical Sciences, C. K. Tedam University of Technology and Applied Sciences (CKT-UTAS), Navrongo, Ghana.

**Keywords:** AVM, DAVFs, endovascular, foramen magnum, transarterial, transvenous

## Abstract

**Rationale:**

Dural arteriovenous fistulas (DAVFs), are rare intracranial arteriovenous malformations which are depicted with direct shunts between meningeal arteries and dural sinuses, dural veins, or cortical veins. We reported a rare case of DAVF in the foramen magnum (FM) which was drained by the jugular veins and the perimedullary veins.

**Patient concerns:**

A 53-year-old male patient was admitted at the Department of Neurosurgery, West China Hospital 5 hours after experiencing a severe headache. His neck was stiff and Brudzinski and Kernigs signs where positive indicating meningeal irritation.

**Diagnoses:**

An emergency computer tomography scan showed subarachnoid hemorrhage while digital subtraction angiography revealed DAVF in the FM.

**Interventions:**

Endovascular therapy which comprised of superselective arteriography and whole brain angiography under general anesthesia was successfully carried out.

**Outcomes:**

Post-embolization angiography showed that the DAVF in the FM area disappeared. Also, whole brain angiography was performed and no other anomalies where detected. The patient recovered with no further neurological deficits and 2 years follow-up revealed the patent is well and go about his daily duties.

**Lessons:**

DAVF in the FM region can be characterized with primary contralateral internal jugular drainage with minor perimedullary involvement.

## 1. Introduction

Dural arteriovenous fistulas (DAVFs), are rare intracranial arteriovenous malformations which are depicted with direct shunts between meningeal arteries and dural sinuses, dural veins, or cortical veins.^[1]^ They accounting for about 10% to 15% of all cranial vascular malformations with annual incidence of approximately 1.044/1,00,000 people.^[2]^ DAVF in the foramen magnum (FM) which occur in the peripheral sinus and condylar veins are rare subtypes that account for only 2% to 5% of all DAVF.^[3,4]^ Computer tomography (CT) scan and magnetic resonance imaging (MRI) may be useful in the detection of DAVF, however, the gold standard radiological modality for detecting DAVFs is digital subtraction angiography (DSA).

Currently, although the treatment of DAVF in the FM is controversial, open surgery for DAVFs in the FM are feasible, but the technique is complex, and associate with high risk. Therefore, interventional therapy is usually regarded as the first choice, and the 2 commonly used interventional modalities are the transvenous (TV) and the transarterial routes. It is worth elaborating that the selection of treatment depends on the vascular structure, location, and venous blood flow direction of the DAVFs. Also, the venous drainage pathway determines the clinical manifestations of DAVFs.^[5]^ DAVFs in the FM have various venous drainage pathways, which are classified according to the Borden and Cognard grading systems.^[6,7]^ High-grade DAVFs are often associated with poor prognosis while DAVFs with perimedullary venous drainage are often considered invasive. We reported a rare case of DAVF in the FM which was drained by the jugular veins and the perimedullary veins.

## 2. Case presentation

A 53-year-old male patient was admitted at the Department of Neurosurgery, West China Hospital 5 hours after experiencing a severe headache. The patient’s headache was primarily located in the occipital region with radiation to the neck region. His headache was associate with painful facial expression, sweating, nausea, and no vomiting. On examination, he exhibited a fixed posture with dilated and round pupils which were dull to light reflex. His neck was stiff and Brudzinski and Kernigs signs where positive indicating meningeal irritation. Also, the World Federation of Neurosurgical Societies scale was II. Preoperative glasgow coma scale was 13 and Modified Rankin Scale score was 3. Routine laboratory investigations were at normal ranges. Electrocardiogram and chest X-ray did not show any abnormities in heart and the chest.

**Figure 1.**
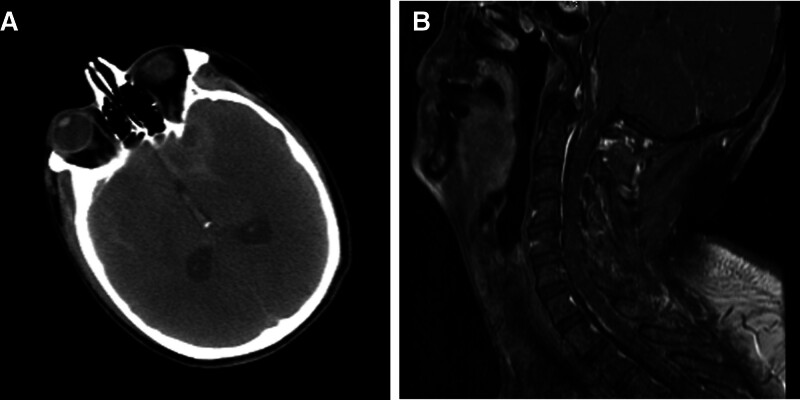
(A) Scattered subarachnoid intracranial hemorrhage shown on emergency CT upon admission and (B) MRI failed to identify the specific cause of the hemorrhage. CT = computer tomography, MRI = magnetic resonance imaging.

**Figure 2.**
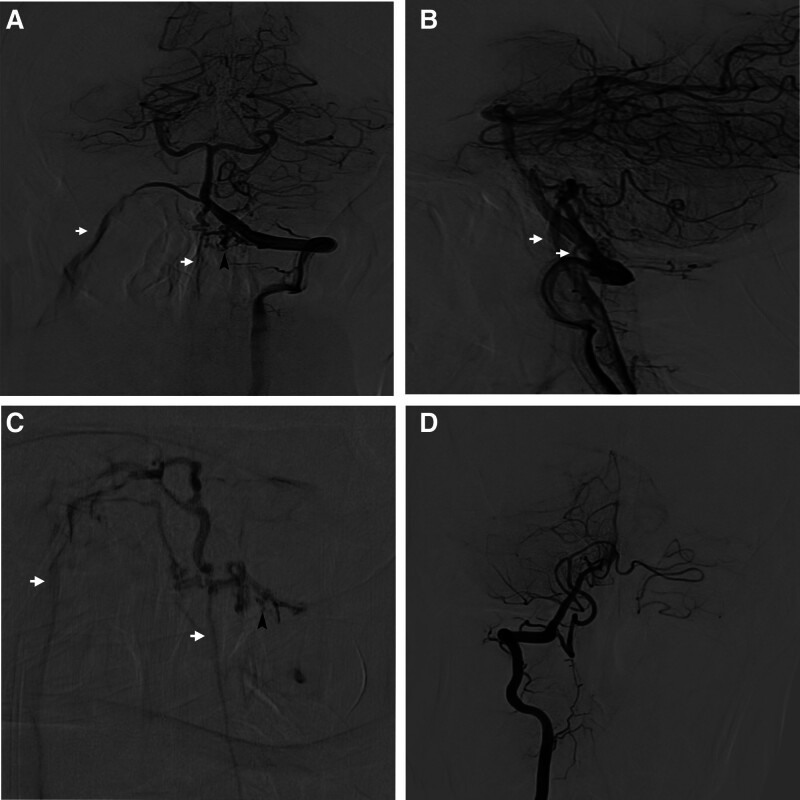
Arteriography showed the anteroposterior (A) and lateral (B) transpondylal images of the arteriovenous fistula. (C) The branch from the left vertebral artery was directly merged into the right internal jugular vein after a tortuously curved section, and a small part of it was incorporated into the perimedullary vein on the way. (D) Is an anterior circulation and right vertebral artery angiography. Note: Black arrow = feeding arteries, white arrow = draining veins.

An emergency CT scan (Fig. 1A) performed on admission showed subarachnoid hemorrhage (SAH). Subsequent cervical MRI only revealed left vertebral artery stenosis and failed to identify the specific cause of the SAH (Fig. 1B). Preoperative DSA revealed DAVF in the FM (Fig. 2A, B). The fistula was drained by the right internal jugular and perimedullary vein constituted by several branches originating from the V4 segment of left vertebral artery (Fig. 2C). A diagnosis of DAVF in the FM and SAH was made, and patient was scheduled for endovascular therapy which comprised of superselective arteriography and whole brain angiography under general anesthesia.

The patient was placed in the supine position, intubated under general anesthesia, and routinely disinfected and draped was carried out. A 6F vascular sheath was inserted into the right femoral artery and a 6F vascular sheath was inserted into the right femoral vein for systemic heparinization under continuous pressure infusion. A loach guidewire guided 6F envoy guide catheter into FM area for cerebral angiography which showed the DAVF. Also, loach guidewire guided 6F envoy guide catheter was introduced into the left vertebral artery. Subsequently, a microguide wire guided microcatheter to the left vertebral artery and microcatheter superselective arteriography showed DAVF and ONXY embolization glue was used to occlude the DAVF. Furthermore, a loach guidewire guided 5F envoy guide catheter was introduce into the right internal jugular vein and microguidewire guided microcatheter introduced in the drainage vein of the DAVF and ONXY embolization glue was used to occlude the DAVF.

**Figure 3.**
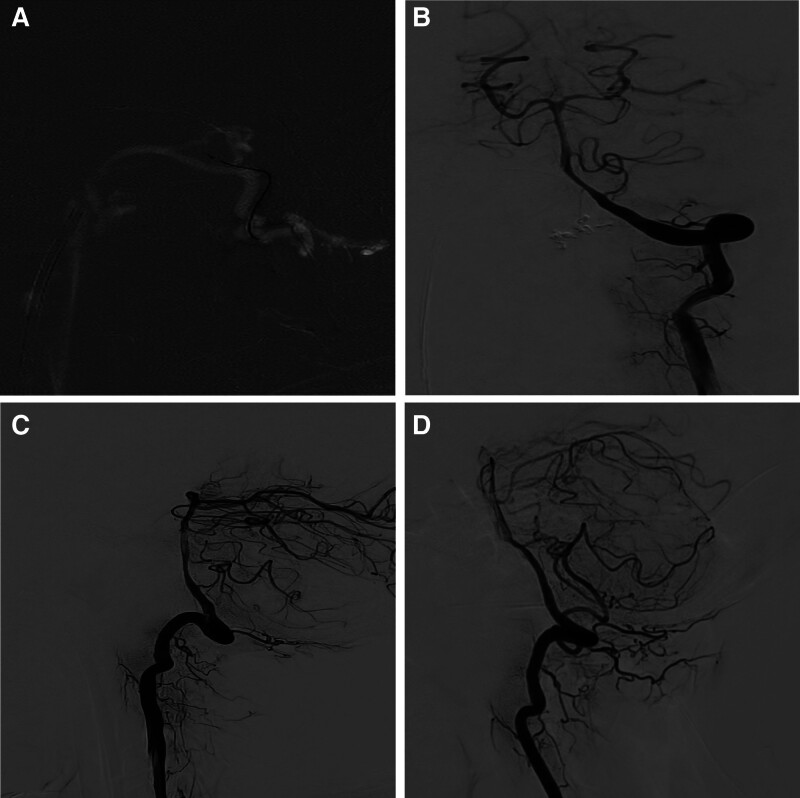
During the embolization process with ONXY glue, (A) the right internal jugular vein was directly superselected to approach the orifice of the fistula by intravenous route, and glue was injected and (B, C) The left vertebral arterial DSA show disappearance of the DAVF in the FM area. (D) Is a DSA at 2 yr follow-up showing no recurrence of the DAVF in the FM area. DAVF = dural arteriovenous fistula, DSA = digital subtraction angiography, FM = foramen magnum.

Post-embolization angiography showed that the DAVF in the FM area disappeared (Fig. 3A–C). Also, whole brain angiography was performed and no other anomalies where detected. The microcatheter, guide catheter, and vascular sheath were withdrawn, and the puncture point was closed with the StarClose SE closure device, with no bleeding. The patient was sent to the anesthesia recovery room after the operation. At discharge, the patients glasgow coma scale was 15 and Modified Rankin Scale score was 0. The patient recovered with no further neurological deficits and 2 years follow-up (Fig. 3D) revealed the patent is well and go about his daily duties.

## 3. Discussion

DAVF is a rare type of intracranial arteriovenous malformations, resulting from abnormal connections between meningeal arteries and venous sinuses, meningeal veins, or cortical veins.^[2]^ This shunt leads to the direct injection of arterial blood into the venous system, bypassing the normal microvascular bed. Consequently, elevated venous pressure and abnormal venous return may occur, potentially associated with factors such as dural sinus thrombosis, venous hypertension, cranial surgery, and trauma.^[8]^ DAVF occurring in the FM region possesses unique diagnostic and classification features. The FM is a stereospatial concept, with its anterior boundary extending from the lower third of the clivus to the upper margin of the axis vertebra, the posterior boundary from the anterior edge of the occipital squama to the spinous process of the axis vertebra, and the lateral boundary from the jugular tubercle to the upper margin of the axis lamina.

The classification of DAVF in the FM remains controversial. Some studies^[9]^ suggest that DAVF in the FM is confined to non-sinus DAVF, where venous backflow occurs directly to the medullary or spinal cord via the bridging veins of the FM. Conversely, sinus DAVF backflow into the rich venous plexus surrounding the FM, including the MS, ACV, suboccipital cavernous sinus, and occipital sinus, does not fit within the narrow definition of DAVF in the FM. Yoo et al categorized medullary bridge venous drainage DAVFs into 9 FM and 13 craniocervical junction lesions.^[10]^ Previously, Li et al classified DAVFs in the FM based on drainage location into anterior condylar canal DAVF, posterior condylar canal DAVF, marginal sinus DAVF, and jugular foramina DAVF.^[11]^ Further classification of the rare DAVFs in the FM is yet to be established. This case represents the first report of a DAVF with direct internal jugular drainage and minor drainage through the perimedullary vein, classified under the FM region.

The clinical manifestations of DAVF are largely determined by its venous drainage pattern.^[12]^ The venous drainage mode of DAVFs in the FM significantly influences treatment plans and patient prognosis. McDougal,^[12]^ Caton,^[13,14]^ and Spittau^[15]^ graded DAVFs in the FM based on drainage patency and reflux. The classifications by Cognard^[7]^ and Borden^[6]^ are the most commonly used, focusing on venous drainage routes. Patients with spinal drainage are classified as Cognard V, a high-grade classification often indicating a poor prognosis. Cognard V cases result in progressive myelopathy in 50% of patients. Venous drainage in the FM can occur towards the cranial or caudal directions, or both. Most lesions at this location drain caudally into the perimedullary vein of the spinal cord are classified as Cognard V. In this case, a rare contralateral internal jugular venous drainage was observed, allowing high-pressure arterial blood to drain directly through the internal jugular vein, thereby avoiding progressive myelopathy. Prior to SAH, no symptoms of pulsatile tinnitus or myelopathy were observed.

The optimal treatment method for DAVF in the FM remains uncertain. Microsurgery, involving techniques such as electrocoagulation, cutting, or clamping to sever arterialized veins, is one approach. However, microsurgery carries significant risks due to the complex anatomy and critical structures of the FM. The deep location of the lesion further complicates the procedure, making it challenging to access without causing collateral damage. Therefore, the balance between effectively treating the fistula and minimizing surgical risks must be carefully considered in each case.^[16,17]^

Currently, endovascular treatment is generally considered the primary choice when indicated, with both TV and TA methods available for angiography and therapy.^[18–20]^ In 12 case reports, Motebejane identified transarterial embolization as the primary treatment for DAVF in the FM. Vijay Madhukar Mundhe, after analyzing 8 cases of DAVF in the FD, emphasized that TA embolization was the preferred route, often achieving complete occlusion with a single TV or combined method.^[5]^ Many researchers also view TV embolization as a superior treatment plan,^[21]^ with Li asserting it as the optimal choice.^[11]^ The approach selection is closely related to the drainage pattern of the DAVF. TA embolization can be challenging when DAVF is supplied by small, highly curved, or multiple bilateral arteries. TV embolization of DAVF involving lesions in the dural sinuses such as cavernous, transverse, and sigmoid sinuses as well as the craniocervical junction is considered safe and effective.^[22]^

The choose of treatment in our case was based on multiple feeding arteries as well as to avoid retrograde flow of ONXY glue into the peri-medullary veins. In this case report, smooth venous drainage made the TV route for embolization superior to the arterial route although both route utilized in the treatment, as the micro-guide wire and micro-catheter could easily reach the fistula opening. However, considering the location of perimedullary vein drainage, we carefully injected ONXY glue before shunting to the perimedullary vein to completely embolize the DAVF while preserving the perimedullary vein. Advances in neurovascular technology, equipment, and embolization materials have facilitated endovascular therapy for treating these lesions. ONXY has become more frequently used among various embolization materials, and multiple studies have shown that ONXY embolization is associated with complete occlusion and lower neurological complications.^[23,24]^ The advantage of ONXY is that when reflux tends to occur, the surgeon can stop until the ONXY glue has solidified and reapplied it, allowing it to flow forward to fill the fistula and the proximal portion of the draining vein.

Finally, for patients with unexplained SAH, cerebrovascular angiographic indications should be appropriately broadened to avoid missing the diagnosis of DAVF that CT and MRI might not indicate. Delayed or misdiagnosed DAVF is not uncommon. In patients with progressive myelopathy as the primary symptom, the initial motor and sensory symptoms can be vague. Early MRI findings are similar to common spinal inflammation, tumors, or compression lesions. The threshold for DSA should be low in highly suspected cases because delayed diagnosis can lead to poor outcomes and disability, even if the fistula is completely occlusive. For patients with SAH, negative CT and MRI can also lead to delays or misdiagnosis, and timely DSA should be performed to identify the cause of the hemorrhage further.

## 4. Conclusion

DAVF in the FM region can be characterized with primary contralateral internal jugular drainage with minor perimedullary involvement. Treatment with intravenous ONXY glue embolization was satisfactory. However, further classification and standardized diagnosis and treatment of DAVF in the FM region require more exploration of its pathophysiology.

## Author contributions

**Conceptualization:** Rui Shang, Ting Wang, Chang-Wei Zhang.

**Data curation:** Rui Shang, Yu-Hu Ma, Si-Hao Li, Ting Wang, Sen Li, Hai-Tao Hu, Seidu A. Richard, Chang-Wei Zhang.

**Formal analysis:** Rui Shang, Yu-Hu Ma, Si-Hao Li, Ting Wang, Sen Li, Hai-Tao Hu.

**Investigation:** Sen Li, Hai-Tao Hu, Seidu A. Richard, Chang-Wei Zhang.

**Methodology:** Rui Shang, Yu-Hu Ma, Si-Hao Li, Ting Wang, Sen Li, Hai-Tao Hu, Seidu A. Richard, Chang-Wei Zhang.

**Software:** Chang-Wei Zhang.

**Writing** – **original draft:** Rui Shang, Seidu A. Richard.

**Writing** – **review & editing:** Rui Shang, Yu-Hu Ma, Si-Hao Li, Ting Wang, Sen Li, Hai-Tao Hu, Seidu A. Richard, Chang-Wei Zhang.
